# Timing and causes of death in septic shock

**DOI:** 10.1186/s13613-015-0058-8

**Published:** 2015-06-20

**Authors:** Fabrice Daviaud, David Grimaldi, Agnès Dechartres, Julien Charpentier, Guillaume Geri, Nathalie Marin, Jean-Daniel Chiche, Alain Cariou, Jean-Paul Mira, Frédéric Pène

**Affiliations:** Réanimation médicale, Hôpital Cochin, 27 rue du Faubourg Saint-Jacques, 75014 Paris, France; Faculté de Médecine, Université Paris Descartes, Sorbonne, Paris Cité, France; Institut Cochin, INSERM U1016, CNRS UMR8104, Paris, France; Centre d’épidémiologie et de recherche clinique, Hotel-Dieu, AP-HP, Paris, France

## Abstract

**Background:**

Most studies about septic shock report a crude mortality rate that neither distinguishes between early and late deaths nor addresses the direct causes of death. We herein aimed to determine the modalities of death in septic shock.

**Methods:**

This was a 6-year (2008–2013) monocenter retrospective study. All consecutive patients diagnosed for septic shock within the first 48 h of intensive care unit (ICU) admission were included. Early and late deaths were defined as occurring within or after 3 days following ICU admission, respectively. The main cause of death in the ICU was determined from medical files. A multinomial logistic regression analysis using the status alive as the reference category was performed to identify the prognostic factors associated with early and late deaths.

**Results:**

Five hundred forty-three patients were included, with a mean age of 66 ± 15 years and a high proportion (67 %) of comorbidities. The in-ICU and in-hospital mortality rates were 37.2 and 45 %, respectively. Deaths occurred early for 78 (32 %) and later on for 166 (68 %) patients in the ICU (*n* = 124) or in the hospital (*n* = 42). Early deaths were mainly attributable to intractable multiple organ failure related to the primary infection (82 %) and to mesenteric ischemia (6.4 %). In-ICU late deaths were directly related to end-of-life decisions in 29 % of patients and otherwise mostly related to ICU-acquired complications, including nosocomial infections (20.4 %) and mesenteric ischemia (16.6 %). Independent determinants of early death were age, malignancy, diabetes mellitus, no pathogen identification, and initial severity. Among 3-day survivors, independent risk factors for late death were age, cirrhosis, no pathogen identification, and previous corticosteroid treatment.

**Conclusions:**

Our study provides a comprehensive assessment of septic shock-related deaths. Identification of risk factors of early and late deaths may determine differential prognostic patterns.

## Background

The incidence of sepsis is increasing as a result of population’s aging and associated comorbidities such as cancer, immunosuppression, diabetes mellitus, or chronic organ dysfunctions. Although the higher number of cases accounted for an overall increase in sepsis-related deaths, the attributable fatality rate significantly dropped at the turn of the century. About 20 years ago, the crude mortality rate of septic shock exceeded 50 % in non-selected populations [1, 2]. Since then, improvement in management of severe sepsis and septic shock translated into increased survival rate in observational studies [3–8]. This is also emphasized by the low mortality rates ranging from 24.2 to 32 % observed in the control arms of recent interventional studies on severe sepsis and septic shock [9–11].

While the literature is plenty of studies with vital status as the main outcome, it is striking that very few data are available about the time to death and the causes that ultimately led to death in septic patients. Nowadays, the combination of anti-infective treatments and aggressive organ failure supports often allows stabilization of the clinical condition, but patients become then exposed to intensive care unit (ICU)-acquired complications that significantly impact on the prognosis. Nevertheless, most epidemiological and interventional studies about septic shock report crude mortality rates that make deaths directly related to the initial septic process or to secondary nosocomial infections hardly distinguishable. The negative results of interventional studies in sepsis despite a robust scientific rationale should then question about the pathophysiological mechanisms leading to death of septic patients. Lastly, a number of ICU patients die after therapeutic limitations as a result of withholding or withdrawing life support decisions that were not accurately reported within studies. With respect to the current trends in epidemiology and outcome of septic shock and to the design of future interventional trials in the field, a better comprehension of the clinical course of septic shock is required beyond the common but gross endpoint of vital status. To this aim, we performed a retrospective study aimed at studying the timing and related causes of death in this setting, as well as respective predictors of early and late deaths.

## Methods

### Patients and setting

The study took place in a 24-bed tertiary medical ICU with an average of 1600 admissions per year. From January 2008 to July 2013, all adult patients (age ≥18 years old) diagnosed with septic shock within the first 48 h of ICU admission were included. Septic shock was defined by the association of the following: (i) a suspected or proven infection, (ii) at least two criteria of systemic inflammatory response syndrome, (iii) an acute circulatory failure requiring vasopressor support. There were no exclusion criteria other than age below 18 years old. Septic shock was defined as a microbiologically proven or clinically suspected infection, associated with acute circulatory failure requiring vasopressors despite adequate fluid filling [12]. Senior staffing remained stable over the 6-year study period. End-of-life decisions to withhold or withdraw life support were taken on collectively when maintenance or increase of life-sustaining therapies was considered as futile by all participants and that death would irremediably occur in a short-term manner. This procedure is standardized in our unit. Data were computed from medical files and extracted from the patient data management system (Clinisoft, GE Healthcare). The ethics committee of the French Intensive Care Society (Société de Réanimation de Langue Française, CE SRLF #12-402) approved the protocol and waived patient’s consent.

### Intended care for patients with septic shock

Patients were promptly treated with broad-spectrum antibiotic combination, depending on the site of infection, previous antibiotic treatment, and known colonization with multidrug-resistant bacteria. Antimicrobial treatment was deescalated to narrower spectrum after identification of the responsible pathogen. Source control measures, such as surgery or removal of infected devices, were applied when necessary. Besides, patients were treated according to the 2008 guidelines of the Surviving Sepsis Campaign [13]. Renal replacement therapy was commonly indicated in established acute renal failure but was also performed early in the course of septic shock in case of profound metabolic acidosis.

### Collection of data

For each patient, the following data were collected: demographics (age and gender), comorbidities, malignancy under treatment within the year prior to ICU admission. Features of immunosuppression included intravenous chemotherapy during the last 3 months, leuconeutropenia defined by leucocyte count <1000/mm^3^ and/or neutrophil count <500/mm^3^, HIV infection at any stage, treatment with corticosteroids for more than 3 months at any dosage, or ≥0.5 mg/kg prednisone equivalent per day for more than 7 days, and treatment with other immunosuppressive drugs.

Characteristics of the primary infection were described as follows: community- or healthcare-acquired, source of infection, bacteremia, causing pathogen. The adequacy of initial antibiotic treatment was assessed in microbiologically documented infections and was defined as at least one antimicrobial active against the pathogen. Severity at admission was assessed by Simplified Acute Physiology Score II (SAPS II) and Sequential Organ Failure Assessment (SOFA) scores during the first 24 h of ICU admission [14, 15].

### Characteristics of deaths

The modalities of death were investigated by collecting the date, the location (in-ICU and in-hospital), and the main cause of death. Early and late deaths were a priori defined as occurring within or after 3 days following ICU admission, respectively. This was based on previous works performed in non-cancer critically ill patients and cancer patients with septic shock, for whom trends in organ failures during the first 3 days in the ICU were found accurate predictors of outcome as compared to single assessment on admission [16–18, 8]. Hence, it is our usual policy of full engagement for a 3-day time frame for most septic patients. Allocation of the cause of death was aimed to identify the disease responsible for fatality rather than the final common pathways of terminal organ failures. Disorders responsible for death were determined by analysis of the medical file by two independent physicians (FD and DG), and discrepancies were resolved by consensus. ICU-acquired infections were defined as new infections acquired more than 48 h after ICU admission. Decisions to withholding or withdrawing life support were also collected. Mesenteric ischemia was proven by macroscopic exam during surgery or by digestive endoscopy or was strongly suspected on the basis of clinical symptoms, elevated arterial blood lactate, and/or biological evidence of acute cell lysis (elevated serum levels of lactate dehydrogenase, creatine phosphokinase, and transaminases) combined to suggestive abdominal CT scan findings such as lack of contrast enhancement of the bowel, pneumatosis intestinalis, and pneumatosis in the intra-hepatic portal vein branches [19].

### Statistical analysis

Continuous variables were expressed as mean ± SD or median (interquartile range) and categorical variables as numbers and percentages. The characteristics of patients who died early (≤3 days from ICU admission), lately (>3 days), or who survived were compared using chi-square tests or Fisher exact tests as appropriate for categorical variables and ANOVA for continuous variables. Factors independently associated with early or late in-hospital death were identified using a multinomial logistic regression model using the status alive as the reference category. Variables significantly associated with early or late death in univariate analysis were included in the model. Multicolinearity was checked by calculating the variance inflation factor. A *p* < 0.05 was considered statistically significant. Statistical analyses were performed using SAS version 9.3.

## Results

### Patients’ characteristics

**Fig. 1:**
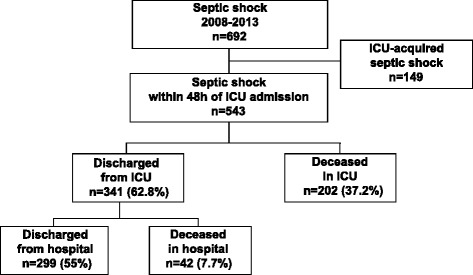
Flow chart of the study. Percentages were computed from the study cohort of 543 patients

During the study period, 543 patients presented with septic shock during the first 48 h following ICU admission (Fig. 1 and Table 1). Sixty-seven percent of patients had underlying comorbidities and 40 % were immunocompromised. Healthcare-associated infections accounted for 53 % of septic episodes. The main site of primary infection was the lung (53 % of patients). A causing pathogen was identified in 69 % of patients. With respect to organ failure supports, invasive mechanical ventilation and renal replacement therapy were required in 88 and 51 % of patients, respectively.

**Table 1:** Characteristics of survivors and early (≤3 days) and late (>3 days) deceased

Variables	All patients	Alive	Early deaths	Late deaths	*p*
	*n* = 543	*n* = 299	*n* = 78	*n* = 166	
Demographics					
Age, years	66 ± 15	63 ± 16	70 ± 15	69 ± 13	<0.0001
Male gender	346 (64)	196 (66)	49 (63)	101 (61)	0.64
Comorbidities					
Malignancy	139 (25)	61 (20)	32 (41)	46 (28)	0.0008
Chronic heart failure	126 (23)	59 (20)	18 (23)	49 (29)	0.06
Chronic pulmonary disease	103 (19)	47 (16)	15 (19)	41 (25)	0.06
Diabetes mellitus	96 (18)	46 (15)	22 (28)	28 (17)	0.03
Cirrhosis	65 (12)	28 (9)	9 (11)	28 (17)	0.06
Chronic kidney failure	60 (11)	29 (10)	8 (10)	23 (14)	0.38
Factors of immunosuppression					
Chemotherapy during the last 3 months	64 (12)	33 (11)	13 (17)	18 (11)	0.36
Leuconeutropenia	41 (7)	21 (7)	13 (17)	7 (4)	0.002
Corticosteroid treatment	91 (17)	39 (13)	15 (19)	37 (22)	0.03
Other immunosuppressive drugs	46 (8)	19 (6)	8 (10)	19 (11)	0.14
HIV infection	6 (1)	4 (1)	0 (0)	2 (1)	0.86
Infection characteristics					
Healthcare-related infections	286 (53)	148 (49)	44 (56)	94 (57)	0.26
Source of infection					0.003
Lung	286 (53)	146 (49)	43 (55)	97 (58)	
Abdominal	91 (17)	40 (14)	15 (19)	36 (22)	
Urinary tract	63 (12)	46 (15)	6 (8)	11 (7)	
Catheter	18 (3)	15 (5)	1 (1)	2 (1)	
Skin and soft tissue	33 (6)	22 (7)	2 (3)	9 (5)	
Others^a^	52 (9)	30 (10)	11 (14)	11 (7)	
Microbiological documentation	376 (69)	221 (74)	48 (61)	107 (64)	0.03
Type of microorganism^b^					0.50
Gram-negative bacteria	206 (55)	127 (58)	26 (54)	53 (49)	
Gram-positive bacteria	151 (40)	82 (37)	21 (44)	48 (44)	
Others^c^	19 (5)	11 (5)	1 (2)	7 (6)	
Positive blood cultures	166 (30)	102 (34)	22 (28)	42 (25)	0.12
Admission biological findings					
Leucocytes (G/L)	12.0 [5.6–18.7]	11.3 [6–18.4]	9.2 [1.7–25]	13 [6.7–18]	0.07
Arterial blood lactate (mmol/L)	2.9 [1.5–5.4]	2.2 [1.4–4]	7.8 [4.3–11.8]	2.7 [1.4–5.9]	<0.0001
Creatininemia (μmol/L)	139 [88–215]	129 [82–186]	196 [127–281]	137 [85–214]	0.02
Prognostic scoring systems					
Admission SOFA score	9 [6–12]	9 [5–12]	11 [9–14]	9 [5–12]	0.0005
Admission SAPS II	66 [51–85]	58 [46–80]	86 [77–101]	67 [53–83]	<0.0001
Organ failure supports					
Mechanical ventilation	476 (88)	241 (81)	77 (99)	158 (95)	<0.0001
Renal replacement therapy	277 (51)	101 (34)	65 (83)	111 (67)	<0.0001
Stress-dose corticosteroids	250 (46)	111 (37)	58 (74)	81 (49)	<0.0001

Categorical variables are expressed as numbers (*percentages*) and continuous variables as mean ± SD or median [interquartile range] as appropriate. Comparisons were performed between the three subgroups (alive patients and early and late deceased)

*HIV* human immunodeficiency virus, *SOFA* Sequential Organ Failure Assessment, *SAPS* Simplified Acute Physiology Score

^a^Meningitis (*n* = 7), bone and joint (*n* = 7), endocarditis (*n* = 5), gynecological (*n* = 4), mediastinitis (*n* = 3), head and neck (*n* = 2), unknown (*n* = 24)

^b^Percentages are computed from patients with microbiological documentation

^c^Fungi (*n* = 8), virus (*n* = 9), mycobacteria (*n* = 2)

### Timing and causes of deaths

**Fig. 2:**
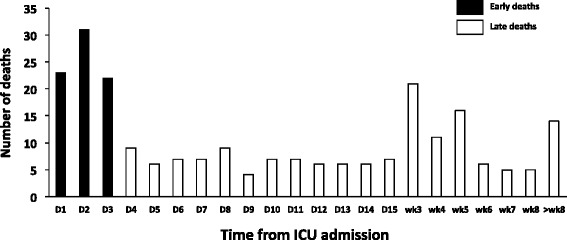
Distribution of deaths according to time from ICU admission. Numbers of deaths are represented per day during the first 2 weeks and per week thereafter. Early (≤3 days) and late (>3 days) deaths occurred in 78 (32 %) and 166 (68 %) patients, respectively

The in-ICU and in-hospital mortality rates were 37.2 and 45 %, respectively, and remained consistent over the study period. Among 244 patients deceased in the hospital, 78 (32 %) died within the first 3 days of ICU admission (early deaths) and 166 (68 %) died thereafter (late deaths) in the ICU (*n* = 124) or in the hospital (*n* = 42) (Fig. 2).

**Fig. 3:**
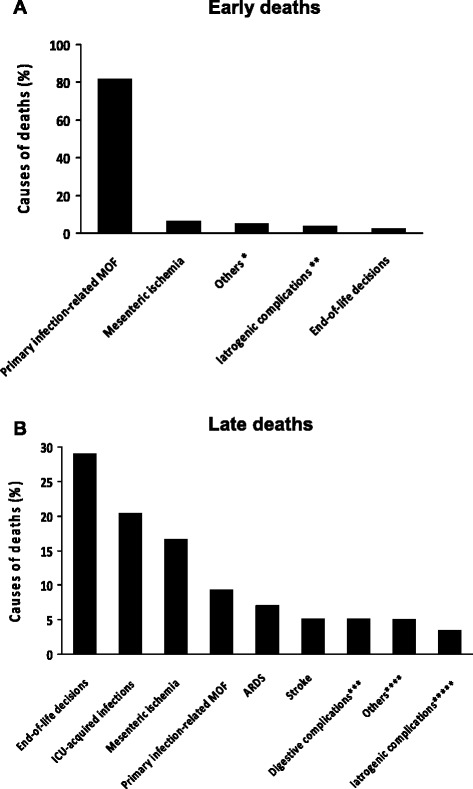
Definite causes of early (**a**, *n* = 78) and late (**b**, *n* = 124) deaths in the ICU. Others (*one asterisk*): myocardial infarction (*n* = 1), pulmonary embolism (*n* = 1), ARDS-related refractory hypoxemia (*n* = 1), intestinal obstruction (*n* = 1). Iatrogenic complications (*two asterisks*): hypoxemic cardiac arrest complicating emergency endotracheal intubation (*n* = 3). Digestive complications (*three asterisks*): gastrointestinal hemorrhage (*n* = 2), digestive perforation (*n* = 3). Others (*four asterisks*): myocardial infarction (*n* = 2), lymphoma-related tumor lysis syndrome (*n* = 2), extensive limb ischemia (*n* = 1), acute liver failure (*n* = 1). Iatrogenic complications (*five asterisks*) related to chest tube insertion (*n* = 1), catheter insertion (*n* = 1), accidental removal of tracheostomy (*n* = 1), and cardiac arrest at the start of hemodialysis (*n* = 1). *MOF* multiple organ failure, *ICU* intensive care unit, *ARDS* acute respiratory distress syndrome

We investigated the primary disorders directly responsible for death in septic patients. With respect to the 42 patients who died after ICU discharge, a comprehensive assessment of the primary cause of death could not be reliably investigated from medical reports. Of note, only six patients who had been transferred to their referent hospital at ICU discharge were readmitted to the local ICU and died there. Definite causes of early and late deaths in the ICU are displayed in Fig. 3. End-of-life decisions precipitated early death in two patients with end-stage comorbidities and previous do-not-resuscitate orders. Late deaths in the ICU also directly resulted from end-of-life decisions in 36 (29 %) patients with fixed multiple organ failure for whom the treating physicians were convinced that meaningful recovery was not possible. Therefore, extensive diagnostic procedures were not carried out in such settings of exclusive palliative care. Besides, most early-onset deaths were directly related to the primary infection through intractable shock and multiple organ failure (82 % of early decedents) (Fig. 3a). In addition, 6.4 % of early decedents were diagnosed with mesenteric ischemia likely to act as a consequence or a contributor of multiple organ failure. Most late deaths were related to ICU-acquired complications such as nosocomial infections (20.4 %) and mesenteric ischemia (16.6 %) (Fig. 3b). Regardless of the time to death, the diagnosis of mesenteric ischemia was proven by surgery (42 %) or endoscopy (5 %) or relied on highly suggestive CT scan findings (53 %).

### Risk factors of early and late deaths

**Fig. 4:**
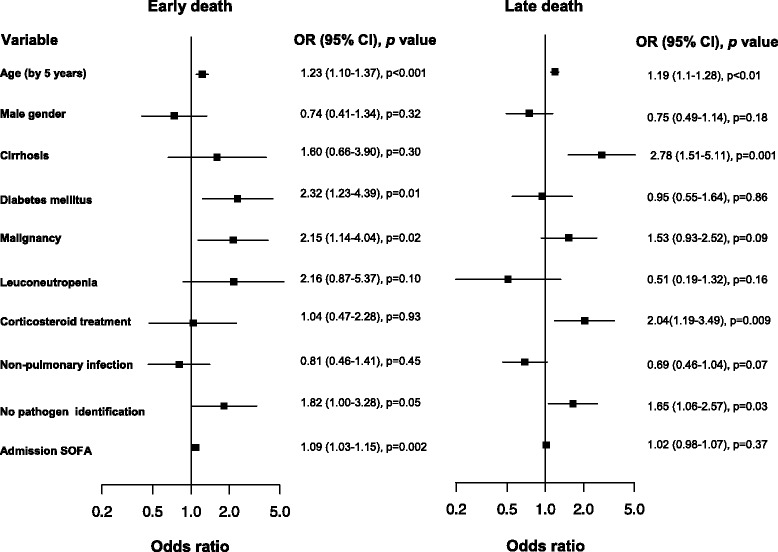
Prognostic factors of early- (≤3 days) and late-onset (>3 days) mortality. Results of the multivariate multinomial logistic regression analysis. All variables entered into the model are displayed on the figure. The variance inflation factor was less than or around 1 for all variables that denotes a low risk of colinearity

We investigated the determinants of mortality with respect to the timing of death by comparing the characteristics of patients who survived or died either early or lately (Table 1). In addition, most patients (93 %) with microbiologically documented infections received adequate initial antimicrobial treatment, without any difference among survivors and early and late decedents (94, 87, and 91 %, respectively, *p* = 0.19). In multivariate analysis, increasing age, diabetes mellitus, malignancy, lack of pathogen identification, and higher admission SOFA score remained independent risk factors of early death. Among patients who survived the first 3 days, independent determinants of late death included increasing age, cirrhosis, lack of pathogen identification, and previous corticosteroid treatment (Fig. 4). When entered into the model, neither chronic pulmonary disease nor chronic heart failure was associated with early or late death (data not shown).

## Discussion

The crude mortality rate is commonly used as the main criterion of outcome in septic shock, but it is clearly limited to assess the prognosis with respect to the clinical diversity of the disorder. Although the visual aspects of survival curves from retrospective or prospective studies provide a gross evaluation of death kinetics in the course of septic shock, specific and reliable data about the timing of deaths are scarce. We herein provided an original comprehensive analysis of modalities of death in septic shock. Because obtaining an accurate etiological diagnosis is of paramount importance in the outcome of critically ill patients, we aimed at describing the primary cause of death as a disease and not as accumulation of organ failures. With respect to the high prevalence of comorbidities and high severity scores on admission, the in-hospital mortality rate of septic shock was 45 % in the present unselected cohort. This is in line with the in-hospital mortality rate of 48.6 % in a recent French cohort of septic shock with similar features, including a similar prevalence of comorbidities (65 %) [5]. The current mortality rate of the ANZICS cohort was reported seemingly lower (22 %), but it encompassed both severe sepsis and septic shock, and the prevalence of comorbidities was only 35 % [4].

The extent of multiple organ failure accounted for the large majority of early deaths in the present cohort. However, the 3-day mortality rate was only 14.3 %, compared to the 3-day mortality rate of 27 % in a cohort of septic shock patients managed in the ICU during the early 1990s [20]. Two French and Spanish multicenter observational studies that included both severe sepsis and septic shock patients in 2001–2002 reported consistent mortality rates of 13.3 and 14.8 %, respectively [21, 22]. Altogether, these data suggest that the overall improvement in the outcome of septic shock is at least partially due to a decrease in the early mortality rate. It is likely that the Surviving Sepsis Campaign bundles for early and aggressive resuscitation of severe sepsis and septic shock contributed to decrease the number of early deaths [23, 13]. Interestingly, admission SOFA scores were similar between alive and late deceased patients, suggesting that late deaths were less related to the initial severity, and were rather dependent on subsequent development of lethal ICU-acquired complications and/or therapeutic limitations. Beyond the classical paradigm of sepsis where overwhelming inflammation is responsible for lethal multiple organ failure, the development of sepsis-induced immunosuppression in patients that survive the primary insult may favor the development of secondary infections in addition to other common risk factors related to severity and requirements for invasive procedures [24–26]. Ventilator-associated pneumonia remains the most dreaded infectious complication in critically ill patients. Although its attributable mortality remains a matter of debate, it is clearly associated with increased duration of mechanical ventilation and increased length of stay in the ICU [27–29].

The identification of mesenteric ischemia as a frequent cause of death both in early and late phases of septic shock is an interesting finding. ICU-acquired mesenteric ischemia is most often associated with microvascular abnormalities. Non-occlusive mesenteric ischemia is a common complication of shock regardless of the underlying etiology and is often considered as the main contributor to terminal multiple organ failure in relation with breaches in the digestive mucosal barrier [30]. Thus, mesenteric ischemia might reflect the severity of shock, and individualizing it as an alternative or independent cause of death is debatable. Yet, the diagnosis is often difficult and is based on the combination of clinical manifestations, biological markers of cell lysis, and suggestive abdominal CT scan findings. A definite diagnosis might be confirmed by digestive endoscopic examination, even up to exploratory laparotomy when highly suspected. In a recent multicenter study of 780 critically ill patients with mesenteric ischemia, the diagnosis was based on CT scan findings in 58 % of patients while it could be proven by surgery and digestive endoscopy in 27 and 15 % of patients, respectively. The outcome was poor as assessed by a mortality rate of 58 % and was dependent on severity but also on the possibility of surgical treatment [19].

Most interestingly, the underlying comorbidities and especially the patterns of immunodeficiency determined differential prognostic patterns in patients with septic shock [31]. Cancer was a predictor of early death, presumably related in part to leuconeutropenia imposed by chemotherapy or bone marrow infiltration. Neutropenia is a major risk factor for fulminant sepsis. In addition, neutropenic sepsis is often associated with non-infectious conditions likely to contribute to mortality in the ICU [32]. Diabetic patients are often affected by chronic organ dysfunctions likely to affect their response to aggression [33]. Altogether, these results highlight the pejorative impact of quantitative or functional defective phagocytosis on early outcome of septic shock. In contrast, patients with previous corticosteroid treatment seemed relatively preserved from early mortality but were more likely to die later. This finding is intriguing but could be related to the impact of corticosteroids on maintenance of vascular response to vasopressors or immunomodulatory properties toward the overwhelming inflammatory response of septic shock [34–36]. Cirrhosis is well known to be associated with a high mortality rate in case of septic shock and was rather found to be associated with late mortality. Aggressive management of septic shock clearly resulted in improved early survival in cirrhotic patients, who then become exposed to secondary infectious and non-infectious complications [37, 38].

This study has several limitations. The major limit certainly lies in the retrospective design of the study. However, we included all consecutive patients within the study period, and the collection of data was almost exhaustive. The characteristics of our cohort, such as the high prevalence (40 %) of immunosuppression, were highly dependent on our hospital environment comprising large departments of oncology, hematology, and internal medicine, and our findings may not fully apply to other centers. In the same way, most patients are admitted to our unit for medical reasons, which limits the generalization to surgical patients. The cut-off between early (≤3 days) and late (>3 days) deaths is debatable but is relevant for refining the prognostic assessment of critically ill patients with septic shock [18, 8]. Furthermore, it allows comparability to previous observational studies [20–22]. In the present study, the distribution of deaths over time clearly exhibited a high mortality rate over the first 3 days, suggesting that this time frame is clinically relevant. We reported the seemingly primary cause of death unambiguously diagnosed at the bedside, but we cannot rule out that some additional disorders might have contributed to the lethal process in such complex clinical settings. A number of patients died in the ICU as a result of end-of-life decisions and therefore did not undergo exhaustive diagnostic procedures. In addition, the primary cause of death of patients deceased after ICU discharge could not be reliably addressed. However, the low rate of ICU readmission (6 out of 42) suggests that most patients had been subjected to therapeutic limitations in the wards. Finally, autopsy was rarely performed in the present cohort because of logistic limitations in our hospital. A number of studies have emphasized that autopsy findings substantially contributed to the diagnosis of critically ill patients [39, 40]. Specifically, an autopsic study in patients who died from sepsis has revealed continuous septic foci that may have contributed to multiple organ failure and death [41].

## Conclusions

We herein provided a comprehensive assessment of septic shock-related deaths. It clearly identifies subgroups of patients with different prognostic patterns in the ICU. Our findings carry several potential implications including early identification of high-risk patients that would require early and aggressive management in the ICU, as well as prevention and detection of secondary complications. In addition, the risk stratification of early or late deaths may also impact on the design and the therapeutic goals of future interventional studies in septic shock.

## References

[1] Alberti C, Brun-Buisson C, Goodman SV, Guidici D, Granton J, Moreno R (2003). Influence of systemic inflammatory response syndrome and sepsis on outcome of critically ill infected patients. Am J Respir Crit Care Med.

[2] Annane D, Aegerter P, Jars-Guincestre MC, Guidet B (2003). Current epidemiology of septic shock: the CUB-Rea Network. Am J Respir Crit Care Med.

[3] Dombrovskiy VY, Martin AA, Sunderram J, Paz HL (2007). Rapid increase in hospitalization and mortality rates for severe sepsis in the United States: a trend analysis from 1993 to 2003. Crit Care Med.

[4] Kaukonen KM, Bailey M, Suzuki S, Pilcher D, Bellomo R (2014). Mortality related to severe sepsis and septic shock among critically ill patients in Australia and New Zealand, 2000–2012. JAMA.

[5] Pavon A, Binquet C, Kara F, Martinet O, Ganster F, Navellou JC (2013). Profile of the risk of death after septic shock in the present era: an epidemiologic study. Crit Care Med.

[6] Levy MM, Dellinger RP, Townsend SR, Linde-Zwirble WT, Marshall JC, Bion J (2010). The Surviving Sepsis Campaign: results of an international guideline-based performance improvement program targeting severe sepsis. Intensive Care Med.

[7] Zuber B, Tran TC, Aegerter P, Grimaldi D, Charpentier J, Guidet B (2012). Impact of case volume on survival of septic shock in patients with malignancies. Crit Care Med.

[8] Pène F, Percheron S, Lemiale V, Viallon V, Claessens YE, Marque S (2008). Temporal changes in management and outcome of septic shock in patients with malignancies in the intensive care unit. Crit Care Med.

[9] Ranieri VM, Thompson BT, Barie PS, Dhainaut JF, Douglas IS, Finfer S (2012). Drotrecogin alfa (activated) in adults with septic shock. N Engl J Med.

[10] Opal SM, Laterre PF, Francois B, LaRosa SP, Angus DC, Mira JP (2013). Effect of eritoran, an antagonist of MD2-TLR4, on mortality in patients with severe sepsis: the ACCESS randomized trial. JAMA.

[11] Caironi P, Tognoni G, Masson S, Fumagalli R, Pesenti A, Romero M (2014). Albumin replacement in patients with severe sepsis or septic shock. N Engl J Med.

[12] Levy MM, Fink MP, Marshall JC, Abraham E, Angus D, Cook D (2003). 2001 SCCM/ESICM/ACCP/ATS/SIS International Sepsis Definitions Conference. Crit Care Med.

[13] Dellinger RP, Levy MM, Carlet JM, Bion J, Parker MM, Jaeschke R (2008). Surviving Sepsis Campaign: international guidelines for management of severe sepsis and septic shock: 2008. Intensive Care Med.

[14] Le Gall JR, Lemeshow S, Saulnier F (1993). A new Simplified Acute Physiology Score (SAPS II) based on a European/North American multicenter study. JAMA.

[15] Vincent JL, Moreno R, Takala J, Willatts S, De Mendonca A, Bruining H (1996). The SOFA (Sepsis-related Organ Failure Assessment) score to describe organ dysfunction/failure. On behalf of the Working Group on Sepsis-Related Problems of the European Society of Intensive Care Medicine. Intensive Care Med.

[16] Timsit JF, Fosse JP, Troche G, De Lassence A, Alberti C, Garrouste-Orgeas M (2001). Accuracy of a composite score using daily SAPS II and LOD scores for predicting hospital mortality in ICU patients hospitalized for more than 72 h. Intensive Care Med.

[17] Guiguet M, Blot F, Escudier B, Antoun S, Leclercq B, Nitenberg G (1998). Severity-of-illness scores for neutropenic cancer patients in an intensive care unit: Which is the best predictor? Do multiple assessment times improve the predictive value?. Crit Care Med.

[18] Larche J, Azoulay E, Fieux F, Mesnard L, Moreau D, Thiery G (2003). Improved survival of critically ill cancer patients with septic shock. Intensive Care Med.

[19] Leone M, Bechis C, Baumstarck K, Ouattara A, Collange O, Augustin P (2015). Outcome of acute mesenteric ischemia in the intensive care unit: a retrospective, multicenter study of 780 cases. Intensive Care Med.

[20] Brun-Buisson C, Doyon F, Carlet J, Dellamonica P, Gouin F, Lepoutre A (1995). Incidence, risk factors, and outcome of severe sepsis and septic shock in adults. A multicenter prospective study in intensive care units French ICU Group for Severe Sepsis. JAMA.

[21] Brun-Buisson C, Meshaka P, Pinton P, Vallet B (2004). EPISEPSIS: a reappraisal of the epidemiology and outcome of severe sepsis in French intensive care units. Intensive Care Med.

[22] Blanco J, Muriel-Bombin A, Sagredo V, Taboada F, Gandia F, Tamayo L (2008). Incidence, organ dysfunction and mortality in severe sepsis: a Spanish multicentre study. Crit Care.

[23] Rivers E, Nguyen B, Havstad S, Ressler J, Muzzin A, Knoblich B (2001). Early goal-directed therapy in the treatment of severe sepsis and septic shock. N Engl J Med.

[24] Hotchkiss RS, Monneret G, Payen D (2013). Immunosuppression in sepsis: a novel understanding of the disorder and a new therapeutic approach. Lancet Infect Dis.

[25] Landelle C, Lepape A, Voirin N, Tognet E, Venet F, Bohe J (2010). Low monocyte human leukocyte antigen-DR is independently associated with nosocomial infections after septic shock. Intensive Care Med.

[26] Grimaldi D, Le Bourhis L, Sauneuf B, Dechartres A, Rousseau C, Ouaaz F (2014). Specific MAIT cell behaviour among innate-like T lymphocytes in critically ill patients with severe infections. Intensive Care Med.

[27] Bekaert M, Timsit JF, Vansteelandt S, Depuydt P, Vesin A, Garrouste-Orgeas M (2011). Attributable mortality of ventilator-associated pneumonia: a reappraisal using causal analysis. Am J Respir Crit Care Med.

[28] Nguile-Makao M, Zahar JR, Francais A, Tabah A, Garrouste-Orgeas M, Allaouchiche B (2010). Attributable mortality of ventilator-associated pneumonia: respective impact of main characteristics at ICU admission and VAP onset using conditional logistic regression and multi-state models. Intensive Care Med.

[29] Valles J, Pobo A, Garcia-Esquirol O, Mariscal D, Real J, Fernandez R (2007). Excess ICU mortality attributable to ventilator-associated pneumonia: the role of early vs late onset. Intensive Care Med.

[30] Wilcox MG, Howard TJ, Plaskon LA, Unthank JL, Madura JA (1995). Current theories of pathogenesis and treatment of nonocclusive mesenteric ischemia. Dig Dis Sci.

[31] Tolsma V, Schwebel C, Azoulay E, Darmon M, Souweine B, Vesin A (2014). Sepsis severe or septic shock: outcome according to immune status and immunodeficiency profile. Chest.

[32] Legrand M, Max A, Peigne V, Mariotte E, Canet E, Debrumetz A (2012). Survival in neutropenic patients with severe sepsis or septic shock. Crit Care Med.

[33] Bertoni AG, Saydah S, Brancati FL (2001). Diabetes and the risk of infection-related mortality in the U.S. Diabetes Care.

[34] Annane D, Sebille V, Charpentier C, Bollaert PE, Francois B, Korach JM (2002). Effect of treatment with low doses of hydrocortisone and fludrocortisone on mortality in patients with septic shock. JAMA.

[35] Keh D, Boehnke T, Weber-Cartens S, Schulz C, Ahlers O, Bercker S (2003). Immunologic and hemodynamic effects of “low-dose” hydrocortisone in septic shock: a double-blind, randomized, placebo-controlled, crossover study. Am J Respir Crit Care Med.

[36] Oppert M, Schindler R, Husung C, Offermann K, Graf KJ, Boenisch O (2005). Low-dose hydrocortisone improves shock reversal and reduces cytokine levels in early hyperdynamic septic shock. Crit Care Med.

[37] Sauneuf B, Champigneulle B, Soummer A, Mongardon N, Charpentier J, Cariou A (2013). Increased survival of cirrhotic patients with septic shock. Crit Care.

[38] Galbois A, Trompette ML, Das V, Boelle PY, Carbonell N, Thabut D (2012). Improvement in the prognosis of cirrhotic patients admitted to an intensive care unit, a retrospective study. Eur J Gastroenterol Hepatol.

[39] Combes A, Mokhtari M, Couvelard A, Trouillet JL, Baudot J, Henin D (2004). Clinical and autopsy diagnoses in the intensive care unit: a prospective study. Arch Intern Med.

[40] Tejerina E, Esteban A, Fernandez-Segoviano P, Maria Rodriguez-Barbero J, Gordo F, Frutos-Vivar F (2012). Clinical diagnoses and autopsy findings: discrepancies in critically ill patients. Crit Care Med.

[41] Torgersen C, Moser P, Luckner G, Mayr V, Jochberger S, Hasibeder WR (2009). Macroscopic postmortem findings in 235 surgical intensive care patients with sepsis. Anesth Analg.

